# Preventive and Remedial Actions in Corporate Reporting Among “Addiction Industries”: Legitimacy, Effectiveness and Hypocrisy Perception

**DOI:** 10.1007/s10551-023-05375-3

**Published:** 2023-03-08

**Authors:** Diletta Acuti, Marco Bellucci, Giacomo Manetti

**Affiliations:** 1 Department of Strategy, Marketing and Innovation, University of Portsmouth, Richmond Building, Portsmouth, PO1 3DE UK; 2 Department of Economics and Management, University of Florence, 9, Via Delle Pandette, 50127 Florence, Italy

**Keywords:** Controversial industries, Addiction, Legitimacy, CSR reporting, Hypocrisy, Effectiveness

## Abstract

The adoption and reporting of CSR policies have important ethical and managerial implications that need scrutiny. This study answers the call of CSR scholars for further studies in controversial sectors by focusing on the voluntary reporting practices of companies that market products or services that generate addiction among consumers. It contributes to the debate on organizational legitimacy and corporate reporting by empirically analyzing whether and how corporations in the tobacco, alcohol and gambling industries disclose their CSR actions and what reactions such disclosures generate in stakeholders. Drawing on legitimacy theory and organizational façades, we apply a consequent mixed-methods design (initiation approach) built on (i) a content analysis of reports prepared by a large set of companies listed on the European, British, US, Canadian, Australian and New Zealand stock exchanges and (ii) an experiment on how different actions taken by the companies (preventive vs. remedial) elicit different perceptions of company hypocrisy and action effectiveness. While previous analyses have focused on “sin” or “harm” industries, this is one of the first to assess how companies account for “addiction”, which is more difficult for them to report and legitimate due to long-term negative consequences. This study contributes to the literature on the instrumental use of CSR reporting by empirically investigating how addiction companies shape their organizational façades and manage organizational legitimacy through disclosure. Moreover, the experimental evidence advances the knowledge of how cognitive mechanisms influence stakeholders in terms of legitimacy assessment and the perceived hypocrisy/effectiveness of CSR disclosure.

## Introduction

In the specialized literature, controversial industries are usually composed of two types of enterprises: “sin” industries, including the alcohol, tobacco, and gambling industries, and other controversial industries involved in emerging environmental, social, and ethical issues, such as defense-related weapons manufacturing, oil and gas production, and hazardous waste (Cai et al., 2011; Hong & Kacperczyk, 2009; Jo & Park, 2020). Sin industries, in particular, are characterized by selling “products, services or concepts which for reasons of delicacy, decency, morality or even fear, arouse reactions of disgust, offense or indignation when mentioned or presented openly” (Wilson & West, 1981, p. 92), but even the consequences of addiction for individual health and family well-being should be considered. Sin industries provoke moral debates, raise problems of an ethical or social nature and are subject to political pressure (Cai et al., 2011). Of course, the definition of controversial industries changes in different historical and cultural contexts (Campbell, 2007; Waller et al., 2005) when the classification criteria adopted are taken into account (Jo & Na, 2012). However, these types of industries generally do not respect basic social rules or behavioral expectations and are therefore perceived as unethical or (partially) illegitimate (Sethi, 1975; Campbell, 2007; Lindgreen et al., 2012; Rest et al., 2012; Jo & Park, 2020).

Among controversial industries, alcohol, tobacco, gambling and online gambling game providers are characterized by products that can create a physical or psychological addiction in consumers according to the Diagnostic and Statistical Manual of Mental Disorders (DSM-5) and the International Classification of Disease, 11th Edition (ICD-11) (APA, 2013; WHO, 2018). The field of addiction has undergone major changes in recent years. Both the DSM-5 and ICD-11 have addressed the nosological question of whether “addiction” should be broadened to include not only psychoactive substances but also specific types of behaviors. The DSM-5 chapter on addictions (“Substance-Related Disorders and Addiction”) lists nine types of substance addictions (alcohol, caffeine, cannabis, hallucinogens, inhalants, opioids, sedatives, hypnotics and anxiolytics, stimulants, and tobacco). A significant novelty of the DSM-5 over the previous version (DSM-4) was the inclusion of gambling disorder in the chapter on substance-related and addiction disorders. In the DSM-4, gambling addiction was placed in the “category of impulse control disorders”. Gambling disorder was included in the addiction disorder following the collection of evidence showing similarities in phenomenology and biology between it and substance use disorders. Additionally, there are unusually high rates of co-occurrence between gambling disorders and substance use disorders (Saunders, 2017).

Given the detrimental effects of “addiction sectors” on society, this research aims to investigate whether and how companies that offer addictive products or services disclose their social responsibility through CSR reports and how stakeholders respond to such communication.

To conduct the present study, we decided to remove from the “addiction sector” all substances that are illegal in most of the analyzed countries (e.g., cannabis, hallucinogens, and inhalants), must be prescribed under medical supervision (e.g., opioids, sedatives, hypnotics, and anxiolytics) or that cannot be diagnosed as a substance use disorder (e.g., caffeine and stimulants) and to add gambling and online gambling disorders, which can be problematic and potentially addictive. Tobacco use Disorder is a diagnosis assigned to individuals who are dependent on the drug nicotine due to use of tobacco products, while alcohol use disorder is a cluster of cognitive, behavioral, and physiologic symptoms indicating that the individual continues using alcohol despite significant alcohol-related problems. With reference to this latter, DSM–5 integrates the two DSM–IV disorders, alcohol abuse and alcohol dependence, into a single disorder called alcohol use disorder (AUD) with the sub-classifications mild, moderate, and severe. According to the ICD-11, gaming disorder is defined as a pattern of behavior characterized by impaired control over gaming, increasing the priority given to gaming over other activities to the extent that gaming takes precedence over other interests and daily activities, and the continuation or escalation of gaming despite the occurrence of negative consequences.

In a large majority of countries in the world, the products and services considered in the present study are legal but still have significant problems of legitimacy because of their ethical, social and health implications for consumers and society at large (Reith, 2007).

In the literature, the adoption of corporate social responsibility (CSR) policies by companies operating in controversial sectors, including those that create addiction through their products or services, is a debated issue that has important ethical and managerial implications (Cai et al. 2011; Geiger & Cuzzocrea, 2017). According to Cai et al. (2011, p. 468), for these companies, the risk of credibility loss and legitimacy due to the morally dubious nature of their products or services (Jo & Na, 2012, p. 442) implies the opportunity, and sometimes the need, to replace CSR practices with regulatory interventions by the state and the legislature. Moreover, governments already exercise regulatory power over gambling, alcohol and tobacco products owing to concessions, licenses, and, more generally, regulations (Deephouse, 1996; Leung & Snell, 2017; Pfeffer & Slancik, 1978), with the primary aim of protecting the health and well-being of citizens and communities (Crane et al., 2004).

This study responds to the call of the specialized literature for research on CSR policies and practices in controversial sectors (Banerjee, 2007; Devinney, 2009; Fatima & Elbanna, 2022; Leung & Snell, 2021) by focusing on companies that market products or services that can generate addiction in consumers and the related voluntary reporting practices. It contributes to the scientific debate on organizational legitimacy through corporate reporting by empirically analyzing whether and how corporations in the “addiction sector” communicate their CSR actions and what the possible intended user reactions are in terms of hypocrisy and effectiveness perceptions.

In the first part of our study, we identified a large set of companies listed on the European, British, US, Canadian, Australian and New Zealand stock exchanges and operating in the “addiction sectors”. More specifically, we investigated their voluntary reporting practices and the actions implemented to counteract harmful effects on the psychophysical health of consumers of their products and services. Thus, in the second part of our study, we conducted an experiment to test whether different actions taken by the companies (preventive vs. remedial) could elicit different perceptions in terms of company hypocrisy and action effectiveness, which in turn affect organizational legitimacy perceptions.

We provide original and up-to-date empirical evidence regarding how companies of the “addiction sector” apply a mix of different but complementary strategies to maintain their legitimacy through CSR reports and jeopardizing policies that have intriguing business ethics implications.

We also obtain interesting results concerning the cognitive mechanisms that lead to legitimacy assessment as stakeholders appear to delegitimize companies when they perceive them to be hypocritical. Furthermore, we find that preventive strategies regarding the negative effects of addictive products and services are perceived to be less hypocritical and more effective than remedial actions to mitigate their harm to consumers.

Our article is structured as follows. The next section presents our conceptual framework, inspired by legitimacy theory. In “Hypothesis development”, we formulate our hypotheses. Then, “Research design” illustrates our methodological approach. “Study 1: Content analysis of nonfinancial disclosure among addiction industries” presents the content analysis of nonfinancial disclosures (Study 1), and in “Study 2: Experiment on effectiveness and hypocrisy perception”, we test our hypotheses through an experiment (Study 2) following an initiation approach. Finally, “Discussion and conclusions” discusses the results of the study and highlights our main conclusions and implications for further research.

## Legitimacy and Hypocrisy in the “Addiction Sector”

According to Suchman (1995), organizational legitimacy is the generalized perception or assumption that the actions of an entity are desirable, proper, or appropriate within a socially constructed system of norms, values, beliefs, and definitions (Suchman, 1995). This definition implies that legitimacy is a desirable social good, that is, something more than a matter of image or perception, and that it may be defined and negotiated at various levels of society (Mitchell et al., 1997). Organizations are thought to be legitimate when they pursue socially acceptable goals in a socially acceptable manner.

Since the production of goods or services that can potentially cause “addiction” in consumers deeply affects an organization’s image, corporations in the “addiction sector” may be tempted to improve or sweeten their legitimacy and credibility in the eyes of stakeholders (Petty & Guthrie, 2000). Remedial actions that counteract the “core business” of a company can be a double-edged sword, as they can appear hypocritical and ineffective in the eyes of stakeholders and may lead to the consequent loss of their own relational capital (Manetti & Bellucci, 2018; Casonato et al., 2019, p. 147; De Castro et al., 2004). Once the license to operate is breached or damaged by illegitimate behaviors, shareholders and funders often sell their shares–causing share prices to plummet–or withdraw their credit lines. Therefore, a good reputation helps to maintain value, whereas a bad reputation tends to destroy it (Gatzert, 2015).

Considering that the majority of the organizations in the “addiction sector” are large corporations that operate internationally and globally and sell controversial products or services, the role of organizational legitimacy (Miller & Michelson, 2013; Rest et al., 2012; Suchman, 1995) is particularly relevant for our study. More specifically, Buhr (1998) presents two dimensions of an organization’s efforts to attain legitimacy: action (whether the organization’s activities are congruent with social values) and presentation (whether the activities appear to be congruent with social values) (Chen & Roberts, 2010). Cho et al. (2015) argue that firms use hypocritical talk, decisions, and actions to manage divergent stakeholder interests and hence maintain legitimacy. The authors refer to “talk” as written or spoken words presenting organizations’ commitments and policies in interaction with the general and competitive environment and, in particular, with external stakeholders. While “decisions” are a special type of talk that indicates a future intention and an increased probability of corresponding actions (Brunsson, 2007), “actions” represent the execution of previous talk and decisions (Brunsson, 1993). Every instance of “talk” contributes to building organizational façades, namely, a symbolic appearance used to manage organizational legitimacy (Abrahamson & Baumard, 2008). According to Abrahamson and Baumard (2008, p. 437), organizational façades are symbolic fronts “erected by organizational participants designed to reassure their organizational stakeholders of the legitimacy of the organization and its management.” An organizational façade can contribute to the creation or reinforcement of organizational legitimacy in the eyes of stakeholders; however, it comprises several facets that serve different roles in managing stakeholders’ diverse expectations. According to Cho et al. (2015), organizational façades and organized hypocrisy can help generate beneficial consequences for many stakeholders despite the incongruence between a corporation’s statements and its actions. For Brunsson (2007), hypocrisy can even present opportunities for change since it can reduce the difference between current and aspirational realities (see also Christensen et al., 2013, p. 385). More specifically, a “progressive façade” aims to show an organization’s progress toward strategic goals, while a “reputational façade” illustrates the organization’s positive image for stakeholders (Abrahamson & Baumard, 2008). In other words, organizations build façades to influence stakeholders’ assessment of their social and institutional practices in an attempt to improve stakeholders’ perceptions of them (She & Michelon, 2019, p. 55).

Legitimacy is also a dynamic concept, as expectations can change over time and particular events might occur that adversely affect the reputation of a company, its legitimacy, and perhaps even its very existence (Makela & Nasi, 2010). Such change is often considered a resource among supporters of legitimacy theory. On the one hand, organizations are dependent on this resource for survival (Dowling & Pfeffer, 1975); on the other hand, organizations can manipulate how society perceives their behavior and activities (Deegan, 2002). Even though companies may attempt to create legitimacy (Sonpar et al., 2010), if consumers, and stakeholders in general, do not accredit it, these CSR efforts could be in vain (Palazzo & Scherer, 2006).

Of course, the concept of legitimacy can be applied to accounting studies and to the reporting practices of organizations. Kuruppu et al., (2019, p. 2081) highlight how legitimating actions relate to subtle, direct, and episodic interventions to placate specific salient stakeholders; these authors find that response behavior is modulated by the need to gain, maintain, or repair legitimacy, especially in intimate interactions with close stakeholders to preserve “good character” and secure the company’s license to operate. As Bebbington et al. (2008) suggest, this sporadic interaction is coherent with the fact that reputation risk management can assist in understanding sustainability or integrated reporting practices and that stakeholder dialog can be used as a mere legitimating tool (Adams, 2004, p.733).

The tension between sustainability discourse and practice has generated extensive and in-depth studies on voluntary corporate sustainability reporting, often resulting in contradictory conclusions (Milne & Gray, 2013; Unerman & Chapman, 2014). While some authors have supported the potential of nonfinancial reporting to make corporations more accountable and transparent about their social and environmental impacts (Bebbington et al., 2014; Rodríguez & LeMaster, 2007), some studies have called into question the validity of this accounting practice because it tends to be limited in scope (O’Dwyer et al., 2005), disingenuous (Aras & Crowther, 2009), and utilized as a legitimacy tool (Cho et al., 2012; Magness, 2006; Milne & Gray, 2007) or to respond to institutional pressures (Thorne et al., 2014). Supporters of legitimacy theory maintain that companies engage in sustainability reporting mainly to secure their own interests (Milne & Gray, 2013) with the explicit aim of deflecting, obfuscating, or rationalizing their relatively poor social and environmental performance under reputational threats (Cho et al., 2010, 2015), rather than indicating rational plans and actions for facing real sustainability problems (Boiral, 2013; Cho et al., 2010; Patten, 2012).

Given the above, we can affirm that legitimacy theory has contributed to accounting and reporting studies in several ways over the past forty years, especially in the field of social and environmental accounting. For example, legitimacy theory is often used to motivate CSR practices and voluntary nonfinancial disclosures. From this perspective, organizations issue nonfinancial reports to reduce their external costs or diminish pressures that are being imposed by external stakeholders or regulators (Adams, 2002; Tate et al., 2010). This behavior occurs because organizations use these reports to influence (or even manipulate) stakeholder perceptions of their image, performance, and impact (Coupland, 2006; Deegan, 2002; Guidry & Patten, 2010). This assertion seems to question the validity of legitimacy theory in stimulating the production of reliable and useful nonfinancial/sustainability reports.

The manipulation of an organization’s image is perceived as being easier to accomplish than improving sustainability performance, supply chain structure, or value systems (Dowling & Pfeffer, 1975). A conscious or unconscious manipulation approach is fairly common among firms (especially the largest ones) that have negative social or environmental impacts, in particular in reporting and communication practices (Lee et al., 2018; Manetti & Bellucci, 2016). For these reasons, legitimacy theory applied to nonfinancial/sustainability reporting is intertwined with stakeholder skepticism within the CSR domain, increasing the idea of corporate hypocrisy and ineffective CSR (Bellucci et al., 2021a, 2021b), especially among consumers and local communities (e.g., Torelli et al., 2012; Wagner et al., 2009). Reporting can be used, in a more or less distorted sense, as a means of demonstrating that an organization has realigned its practices, policies, and performance with the expectations of organizational audiences.

However, manipulation activities can be perceived as hypocritical by stakeholders. Stakeholders’ perceptions of hypocrisy represent a detrimental factor for corporate reputation and, in turn, legitimacy. Hypocrisy occurs when a gap between assertions and actions appears (Shklar, 1984). Thus, hampered accountability presents the risk of engendering accusations of hypocrisy. The latter response occurs in a world in which values, ideas, or people are in conflict and is a means for both individuals and organizations to address such conflict (Brunsson, 2007). In contrast, sincerity is interpreted as a “degree of congruence” that “does not relate to how ethical a company is but how true that company is to its mission statement, value declarations or corporate charter” (Fassin & Buelens, 2011: 587). Glozer and Morsing (2020, p. 365) propose that “conventional definitions of corporate hypocrisy emphasize its basic criterion as a systematic decoupling between talk and action”. In other words, hypocrisy is “the belief that a firm claims to be something that it is not” (Wagner et al., 2009, p. 79). In addition to the interpretation of hypocrisy as behavioral inconsistency, Batson et al., (2006: 321) refer to the “motivation to appear moral yet, if possible, avoid the cost of actually being moral”. Both of these interpretations of hypocrisy–as communication/behavioral inconsistency or deceptive claims–can lead to stakeholders’ negative cognitive responses (Wagner et al., 2020). On the contrary, effectiveness represents the ability to be successful in producing a desired result (Cambridge Dictionary, 2022). Thus, an action is evaluated as effective when it is perceived to achieve the intended performance (Guo et al., 2021). In the context of CSR, perceived effectiveness refers to the degree to which individuals believe that a strategy can help to resolve a problem or generally influence society. It is the perception of the company’s ability to improve important environmental and social issues. While a few studies have begun to explore the connection between hypocrisy perceptions and organizational legitimacy in other contexts (e.g. Bellucci et al., 2021a; Kougiannou & Wallis, 2020), we claim that there is an additional, unexplored, theoretical explanation related to the perceived effectiveness of CSR strategies.

## Hypothesis Development

### Preventive and Remedial CSR Strategies

The statement “prevention is better than cure” is often attributed to the Dutch philosopher Desiderius Erasmus around 1500. Prevention refers to stopping something wrong or dangerous before it happens; cure is dealing with a consequence after it has happened (Scott et al., 2020). This popular phrase is now a fundamental principle of modern social care strategies. In this context, prevention means “stopping problems from arising in the first place; focusing on keeping people healthy, not just treating them when they become ill” (Department of Health and Social Care, 2018, p. 5). Since, according to Brunsson (1993, 2007), “actions” represent the execution of previous discussion and decisions, it is important to verify whether prevention actions can effectively contribute to the reinforcement of legitimacy in companies that try to limit the damages of their addictive business in the attempt to appear less hypocritical and more effective than resorting to remedial actions.

In fact, the economic activities of companies operating in “addiction sectors” are stigmatized as irresponsible, and their legitimacy has often been questioned. Recent studies have shown that companies operating in controversial industries can also be socially responsible despite the contradiction between their business and CSR activities (Oh et al., 2017). What has been questioned regarding gambling, alcohol, and tobacco companies is their impact on society and how they can improve their public image as responsible entities. Therefore, it is important for such companies to design and implement CSR activities that mitigate the stigma related to their production and limit stakeholders’ skepticism regarding their legitimacy.

From a practical point of view, preventive actions are likely to be much more effective and to be received by the public as less hypocritical than attempts to reverse the negative future consequences of harmful products. From a perceptive point of view, communication of preventive strategies may introduce certain negative effects on society but also show how a company can prevent such undesirable outcomes through CSR activities that result in positive outcomes (Kim et al., 2012). Despite the potentially addictive and harmful effects of the products or services offered by these companies–which are socially rejected–the implementation of preventive activities can demonstrate an effective positive impact on society that is socially acceptable. In contrast, limiting the damages of product addiction and finding a remedy a posteriori can be less acceptable and perceived as more hypocritical. Indeed, remedial strategies involve intervening when a negative effect has already occurred. This type of strategy attempts to mitigate damage that should have been avoided with a preventive strategy. This blurred effect of remedial CSR strategies on society makes the organizational activity less legitimate.

Building upon this background, we expect that a preventive CSR strategy can increase stakeholder perceptions of the organizational legitimacy of companies operating in “addiction industries”. In contrast, when companies cure damage, they consciously cause stakeholders to not accredit their legitimacy. Therefore, we propose the following:

#### Hypothesis 1

The CSR preventive strategy increases perceived organizational legitimacy compared to a remedial strategy.

### Hypocrisy Perception

Companies operating in “addiction sectors” struggle to communicate their CSR efforts and convince stakeholders of their genuine concern for consumers’ health (Lindorff et al., 2012). The communication of a preventive vs. remedial CSR strategy can cause different stakeholder responses in terms of hypocrisy assessment and, consequently, organizational legitimacy, as described above. The preventive action of companies involves investing resources to avoid the addictive effects of gambling, alcohol, or tobacco. Thus, the willingness to appear moral aligns with the adoption of responsible action. In this case, the company pursues an acceptable goal in a socially acceptable manner. The remedial action attempts to limit the harm of products that the company deliberately decides to sell. Here, the company enables consumers’ addiction to the products offered to them and then intervenes to remedy the irresponsibility of its business. In other words, the company aims to appear moral, avoiding the cost of actually being moral. Therefore, we expect preventive CSR strategies to elicit a lower perception of hypocrisy compared to remedial strategies. Consequently, in cases of preventive CSR strategies, organizational legitimacy will increase, while in cases of remedial strategies, it will decrease. More formally, we hypothesize the following:

#### Hypothesis 2

The CSR preventive strategy (vs. remedial strategy) decreases hypocrisy perceptions that, in turn, increase organizational legitimacy.

### Perceived Effectiveness

Because of the controversial nature of “addiction industries”, companies still face challenges in building legitimacy among stakeholders through effective CSR initiatives. Thus, they must develop CSR initiatives that effectively address environmental and social challenges to achieve legitimacy among their stakeholders (Devenin & Bianchi, 2018). An effective CSR initiative is capable of producing the intended result for the beneficiaries. When this does not occur, stakeholders do not perceive a real effort of the company in terms of adequate compensation for the impact of its business, and CSR initiatives disclosed by the company will likely be seen as merely an image-cleaning strategy (Clarkson et al., 2011; Sethi et al., 2017). The rationale for a prevention approach is sometimes stated in terms of how intervening at an early stage could reduce the future cost for society. Indeed, it is a more efficient use of corporate and societal resources “to prevent a problem from emerging or to act early than it is to cure the problem or to have to deliver more complex responses” (Kennedy, 2020, p. 354). Hence, we hypothesize that preventive CSR strategies are perceived by stakeholders as more effective than remedial strategies and that the effectiveness of such strategies increases the perceived legitimacy of the company. Conversely, remedial strategies are less effective as they seek to cure damage that has already been caused by the company itself, thereby reducing organizational legitimacy.

#### Hypothesis 3

The CSR preventive strategy (vs. remedial strategy) increases the perceived effectiveness of the strategy, which, in turn, increases organizational legitimacy.

After identifying and analyzing preventive and remedial strategies in corporate reports (Study 1), we test our three hypotheses with a between-subject experiment (Study 2).

## Research Design

In light of the above, in the following sections, we introduce the methodology and results of our two-step study. More specifically, we use the results of the content analysis of corporate reports in the “addiction sector” as a point of departure to further explore stakeholders’ perceptions of perceived organizational legitimacy. Taken together, the two steps of the unitary study offer a more complete picture of the phenomenon being studied and produce robust findings: by collecting and combining the strengths of both qualitative and quantitative data, we provide a better answer to our research question (Hoque et al., 2017).

The content analysis precedes the experiment since we opted for an initiation approach (Davis et al., 2011, p. 469) in which the results of an initial study are used to inform a second study that adopts a different method. The two methods have unequal weights: the less heavily weighted method (content analysis of real reports) is employed to initiate the research and is secondary to the primary method used in the main study (the experiment based on the manipulation of key factors that emerged in the content analysis). Indeed, the content analysis enables us to explore which actions are communicated by companies in the “addiction sector” and to create realistic stimuli for the experiment. The results are reported separately, but the focus of the discussion is on the experiment.

**Fig. 1:**
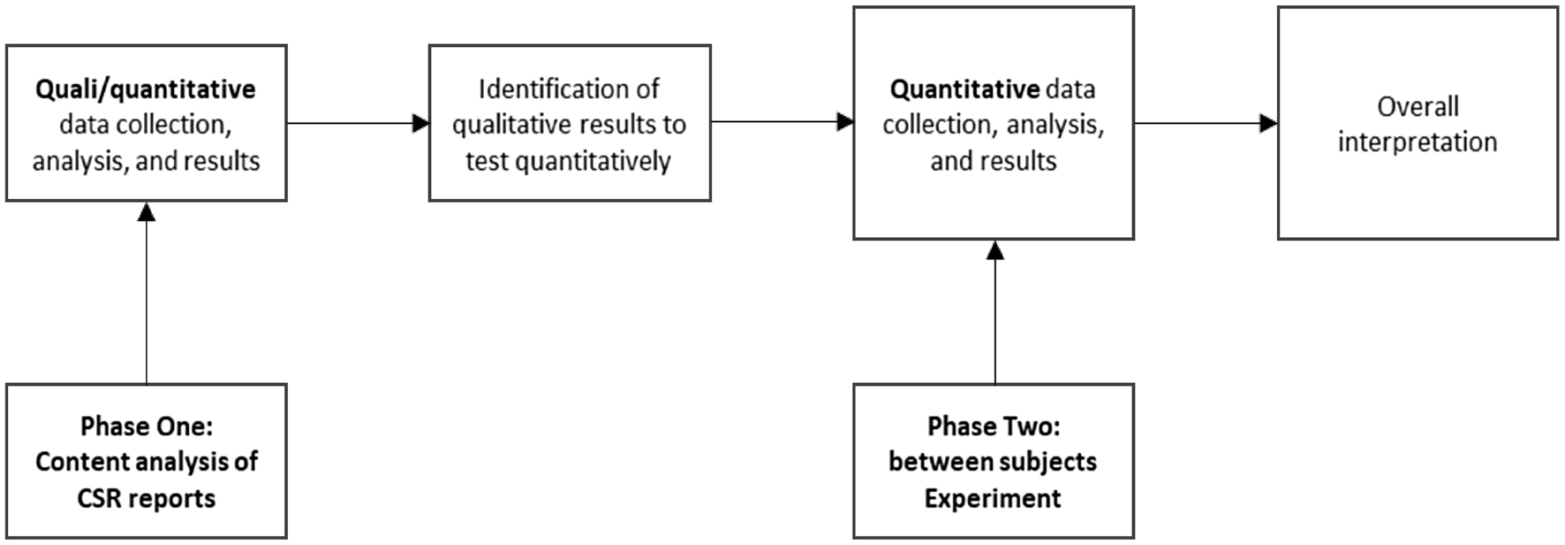
Exploratory mixed methods design

This methodological approach allows us to conduct a preliminary exploration of the phenomenon of remedial actions in corporate reporting (Creswell & Plano Clark, 2007; Tashakkori & Teddlie, 1998). Figure 1 shows the exploratory mixed methods design employed in this research.

## Study 1: Content Analysis of Nonfinancial Disclosure Among Addiction Industries

### Methodology and Data Collection

In the first, exploratory study, we investigate the voluntary reporting practices of an international set of listed companies operating in the “addiction sector” and the reported remedial actions implemented to counteract harmful effects on the psychophysical health of consumers of their products and services.

To this end, we adopted the content analysis methodology, a research technique based on the objective, systematic, and quantitative description of the manifest content of communication (Berelson, 1952). According to Bryman and Bell (2015), content analysis has been increasingly used in business research to examine media items and annual reports. It has been widely adopted in corporate disclosure studies because it allows repeatability and creates valid inferences from the data (Bellucci et al., 2019; Guthrie et al., 2004; Krippendorff, 2004).

As specified in the introduction, our content analysis focuses on the reports of all 162 companies operating in the alcohol, tobacco and online gambling and gaming industries that are listed on the European, British, US, Canadian, Australian and New Zealand stock exchanges. This enabled us to obtain the largest possible set of companies (a) listed in the major EU, US and Commonwealth stock markets, (b) with reports that are expected to be written in English, and (c) with goods and services that, albeit legal in the countries analyzed, are still recognized to create a physical or psychological addiction in consumers according to the Diagnostic and Statistical Manual of Mental Disorders (DSM-5) and to the International Classification of Disease (ICD-11) (APA, 2013; WHO, 2018). Table 1 provides descriptive statistics of the number of included companies by industry and stock market.

**Table 1:** Descriptive statistics by industry and market

Industry	Listed “addiction companies” in each stock market						
	USA	UK	EURO	Australia	Canada	New Zealand	*Totals*
Alcohol	27	11	13	8	5	4	*68*
Gambling	30	9	6	8	4	1	*58*
Tobacco	24	5	4	0	3	0	*36*
*Totals*	*81*	*25*	*23*	*16*	*12*	*5*	*162*

Industry	Listed “addiction companies” in each stock market which provide a standalone or integrated social and environmental report for 2020						
	USA	UK	EURO	Australia	Canada	New Zealand	*Totals*
Alcohol	10	2	10	1	1	0	24
Gambling	10	0	4	1	1	1	17
Tobacco	6	0	2	0	0	0	8
*Totals*	26	2	16	2	2	1	49

Such a content analysis is beneficial in the frame of our research design for evaluating the state of the art and, above all, for identifying realistic stimuli for the experimental study that follows.

As this was an exploratory study based on original data, we first needed to fathom the empirical setting. In the first step of the content analysis, we intended to verify the reporting behavior of the 162 companies mentioned above in 2018, 2019 and 2020. For each year of operation, our content analysis could report three possible kinds of reporting: (1) financial report only, (2) separate financial and sustainability reports, and (3) an integrated report.

Due to the large overlap in the meaning, in this paper, the term “sustainability report” is used to embrace every form of nonfinancial disclosure concerned with social and environmental information, although we acknowledge that “CSR reporting”, “sustainability reporting” and “nonfinancial disclosure” are distinct, albeit correlated, concepts.

The first step of the collection was aimed at separating the companies that voluntarily presented sustainability reports (“2”) or integrated sustainability reports (“3”) from companies that presented only mandatory financial reports (“1”). This first step was conducted through an analysis of the companies’ websites, the interrogation of online databases concerned with sustainability disclosure and, ultimately, a free search using popular web search engines. Through this process, it was possible to identify the set of 49 companies that in 2021 prepared an integrated report or a sustainability report with social or environmental information referring to 2020 as the year of operation. The other 113 companies did not provide a sustainability or integrated report for 2020 and were therefore excluded from the second step of the content analysis.

The sustainability reports of the identified 49 out of 162 companies are the object of the second and main step of our content analysis. To investigate the reporting practices and reported remedial actions of these 49 large companies from the alcohol, tobacco, and online gambling and gaming industries, we developed an analytic framework. This framework is based on a review of the scholarly literature presented in the previous sections and the results of a pilot study conducted in May 2021 in which two expert scholars independently conducted a content analysis of the same preliminary subsample of 12 reports. The results of the pilot study were considered and discussed, and a final set of categories was then defined. In cases of uncertainties or conflicts, the issues were discussed among the whole research team and resolved in a way that would enable the content analysis to obtain richer findings and useful data for Study 2.

Thus, we identified two levels of categories. The first level distinguishes between the preventive or remedial purpose of a company’s action. We defined these two purposes as the objective goals of the company action. When the action purpose is to prevent a problem or effect, there is an objective “*to keep from occurring; avert; hinder”,* and when the action purpose is to cure a problem or effect, there is an objective *“to relieve or rid of something detrimental*” (Scott et al., 2020). The second level pertains to data on whether the different reports address the issue of addictions, whether responsible ways of consumption are promoted, whether strategies that encourage the reduction of consumption of certain products are presented, whether actions taken to mitigate the negative effects of consuming the products include changes to the company’s business model, whether the company takes responsibility for the effects of the consumption of its products, and whether the reports mention any remedies that the company has implemented to counter the negative effects of consumption. The complete set of categories used in our main content analysis is shown in Table 2.

**Table 2:** List and description of data collected from report content analysis (categorized through the analytical framework)

Categories	Motivations for inclusion	Examples of operationalization	Possible outcomes
General			
Addiction issues Does the report address the issue of addictions?	We contribute to the scientific debate on hypocrisy and legitimacy in corporate reporting by empirically analyzing whether corporations in the “addiction sector” communicate their negative impacts and remedial actions	“addict*”, “habit”, “dependence”	“0” not mentioned; “1” mentioned; “2” mentioned in plain sight (e.g., cover, full page, in the letter from the CEO, in a specific section)
Strategy: preventive			
Responsible consumption Are responsible ways of consumption promoted in the report?	A discussion of where corporate responsibility ends and consumer/stakeholder responsibility begins	The slogans “drink responsibly” and “play responsibly”	“0” not mentioned; “1” mentioned; “2” mentioned in plain sight (e.g., cover, full page, in the letter from the CEO, in a specific section)
Demarketing Are strategies that encourage the reduction of consumption of certain products presented in the report?	A reflection on demarketing (Comm, 1998), an attempt by the firm to discourage all or some of its customers from making purchases either temporarily or permanently	The cover of the Philip Morris CSR report 2019 that claims “Progress toward a world without cigarettes”	“0” not mentioned; “1” mentioned; “2” mentioned in plain sight (e.g., cover, full page, in the letter from the CEO, in a specific section)
Changes to business model Do actions taken to mitigate the negative effects of consuming the products include changes to the company’s business model?	Constrained by growing legitimacy issues, companies in the alcohol, tobacco and gambling industries could be led to seek new business opportunities by opening new markets or reconsidering their business models	Stop selling certain products or services, restrict certain markets, present new “safer” product lines (e.g., zero-alcohol beers or electronic cigarettes), etc	“0” no; “1” yes
Strategy: remedial			
Assumption of responsibility Does the company take responsibility in the report for the effects of the consumption of its products?	The addictive aspects of products and services in certain industries have often been overlooked: what are the consequences of this issue on companies’ “walk and talk”? Understanding of whether companies are willing to admit their responsibility for negative impacts on consumers	“We are clear that combustible cigarettes pose a serious health risk” “We use analytical techniques to identify early signs of gambling disorders”	“0” not mentioned; “1” mentioned; “2” mentioned in plain sight (e.g., cover, full page, in the letter from the CEO, in a specific section)
Remedial actions Does the report mention any remedies that the company has put in place to counter the negative effects of the consumption of its products?	CSR in addiction industries is often transactional, but it is difficult to find transformational CSR activities due to the nature of such businesses. Transactional remedies do not involve particular change to the business model	Awareness campaigns, funds for aid centers, etc	“0” not mentioned; “1” mentioned; “2” mentioned in plain sight (e.g., cover, full page, in the letter from the CEO, in a specific section)
Additional elements			
Dialogic stakeholder engagement Are there elements that go beyond purely informative communication (one-way) and that come close to dialogic accounting elements toward stakeholders?	A reflection on stakeholder engagement and materiality analysis processes underlying the reports. Willingness to understand whether reporting is used to establish a dialogic, inclusive, transparent connection with customers and other stakeholders	Presence of parts of the report illustrating CSR actions implemented in response to stakeholder requests (remedial communication strategy) or CSR actions that require stakeholder involvement (involving communication strategy)	“0” not mentioned; “1” mentioned; “2” mentioned in plain sight (e.g., cover, full page, in the letter from the CEO, in a specific section.)
Negative images Does the report contain visual elements with content that refers to negative effects of the products or services?	Although it has been acknowledged that companies prefer messages with a positive valence to promote their social and environmental responsibility, visuals with a negative valence can represent a sign of transparency and credibility (Invernizzi et al., 2022)	E.g., pictures depicting negative health effects of tobacco and alcohol consumption or gambling disorders	“0” not mentioned; “1” mentioned; “2” mentioned in plain sight (e.g., cover, full page, in the letter from the CEO, in a specific section)

Except for the changes to business models (Amit & Zott, 2012; Wirtz et al., 2016) that employ a dichotomous variable, the possible outcomes for each category are as follows: “not mentioned” (e.g., when the report does not mention any remedies that the company has put in place to counter the negative effects of the consumption of its products); “mentioned” (e.g., when the company takes direct responsibility in a single line of the report for the effects of the consumption of its products); and “mentioned in plain sight” (e.g., when the reports spend a whole section to encourage the responsible way of consumption or the reduction of consumption of certain products). The latter outcome is also coded when the category is addressed on the cover of the report, using a full page, through a large-format visual, in the letter from the CEO, or in a specific section that appears in the summary of the report or other highly visible parts of the document.

Thus, the unit of the content analysis is each report, which has been read and manually coded line-by-line. A keyword search was subsequently conducted to ensure that no significant paragraph was overlooked.

The content analysis required a research team composed of an analyst and two scientific supervisors. Specific guidelines were defined and used by the research team. In particular, a list of detection and classification rules based on the categories illustrated above was established and discussed among the members of the research team, and the classification criteria for each category were subsequently identified. Afterward, a preliminary test of the results of the coding procedure was conducted in a second pilot as a means of highlighting ambiguous or unclear coding rules and to standardize the classification capabilities of the researchers. This second pilot was conducted in June 2021 and included 8 reports. The results of the individual classification by the analyst were discussed with the two supervisors. In cases of uncertainties or confusing codes, the issues were once again discussed among the whole research team and resolved in a way that would lead to trustworthy results. These preliminary activities supported the definition of the final set of detection and classification rules that led to the results discussed in the following section.

## Results

In 22 cases out of 49, disclosure was provided by combining an annual report with a CSR/sustainability/ESG report, while in 27 cases, the company offered an integrated report. All the analyzed reports were written in English, published in 2021 and contained information related to 2020.

Appendix 1 illustrates all the quantitative results of our content analysis concerning the research questions and categories specified in the previous section.

We found that only 13 reports (26.53%) addressed the topic of addiction, communicating potential negative impacts and actions the company would take to counter addiction issues. The proportion of companies disclosing information about addiction was higher in the tobacco industry (50%, 4 companies out of 8), where the consequences of product dependence have been clinically recognized for a long time, and lower in the gambling industry (17.65%, 3 out of 17). Only 3 reports from the tobacco industry and one report from the gambling industry addressed the topic of addiction in plain sight with highly visible content, for a total of 8.16% of the reports.

In most cases, companies used their reports to encourage responsible consumption of their products or services, which is the most typical preventive strategy to maintain legitimacy. In 26 cases, the preparers of the report dedicated clearly visible content (e.g., a large title, special section, letter from the CEO, cover) to promoting responsible consumption of the company products, implicitly admitting that nonmoderate use could lead to physical or psychological harm. In the other 12 cases, content on this issue was present but located in less prominent parts of the reports. Overall, responsible consumption was promoted by 75% of the reports for the alcohol industry (e.g., “drink responsibly”, “don’t drink and drive”), 94.12% for the gambling industry, and only 50% for the tobacco industry.

Our content analysis found potential traces of demarketing strategies in 16 reports out of 49; these contents could signal an attempt by the firm to discourage all or some of its customers from making purchases either temporarily or permanently (Comm, 1998). It is interesting to highlight how this behavior was found to be unusual in the gambling industry (11.76%) and sporadic in the alcohol industry (29.17%) but predominant in the tobacco industry (87.50%). This behavior can be explained by the fact that the negative effects of smoking on health are now manifest, and tobacco companies are turning to (as they are being forced to) the sale of different “reduced-risk” products that do not burn tobacco to deliver nicotine to the user. The emphasis of tobacco companies in their reports on the development of diverse business models appears to function as part of their organizational façades used to create an impression of organizational legitimacy (Abrahamson & Baumard, 2008; Cho et al., 2015). Only 3 reports from the tobacco industry and 2 reports from the alcohol industry addressed the topic of consumption reduction in plain sight with highly visible content, for a total of 10.2% of the reports.

The same motivation underlies the highest willingness shown by tobacco companies to clearly take responsibility in the reports for the negative effects of the consumption of their products. Half of the tobacco companies addressed their responsibility in the report, and one-third (37.5%) also used clearly visible content in plain sight. However, only 29.17% of the alcohol companies and 11.76% of the gambling companies used their reports as an accountability mechanism to clearly state their responsibility; this was probably because the negative impacts of alcohol and gambling in terms of addiction and psychological and physical health are still less socially constructed, although they have been confirmed from a medical standpoint (APA, 2013; WHO, 2018).

Many of the companies under observation (57.14%) used their reports to mention remedies that they had implemented to counter the negative effects of the consumption of their products; however, this information was not always in plain sight with highly visible content (32.65%). It is interesting to highlight that companies in the alcohol and gambling sector, which, as we have just noted, appear reluctant to disclose their preventive responsibility for the negative effects of their products, were instead proactive in showing commitment to the implementation of remedial actions (66.67% and 64.71%, respectively). In contrast, tobacco companies, practically obliged, for institutional and legal reasons, to declare in advance their responsibilities regarding the evident negative effects of their products, appeared reluctant to report remedial actions. It appears that tobacco companies are forced to follow a path toward a “progressive façade” and to show their progress toward strategic goals, while gambling companies can limit their efforts to a “reputational façade” that focuses on the organization’s positive images for stakeholders (Abrahamson & Baumard, 2008). Alcohol companies appear equidistant, promoting a “responsible consumption” that also serves as disclaimer for their accountability (responsibilities are partially accounted for and partially delegated to stakeholders).

At this point, it is interesting to try to understand whether and how the growing legitimacy problems around the products of “addiction companies” are influencing changes in their business models and, particularly, how a company’s business model is reported in its nonfinancial disclosure. Corporate social responsibility in “addiction industries” is often transactional (where the rationale of engagement is to adapt to the strategic opportunities sensed in the environment), but it is uncommon to find transformational CSR activities (guided by deeply held moral direction) (Castelló et al., 2009). Remedies such as donations to research and aid institutions do not involve particular changes to the business model. Our results for the gambling industry, which appeared to focus on remedial actions and keeping business as usual, confirmed this scenario. However, our content analysis also indicated that many alcohol companies and, remarkably, tobacco companies did report their actual actions or future intention to reshape or expand their business model around “alcohol-free” and “tobacco-free” products (58.3% and 75%, respectively). This means that they invested significant resources to design, produce and promote new products that were less harmful for consumers’ health and that could replace traditional products. For example, the aim of alcohol-free beer is to reproduce the taste of beer while eliminating the inebriating, addictive effects of standard alcoholic brews. Another example is tobacco-free cigarettes, which do not contain any tobacco or nicotine, thereby limiting the nicotine addiction, while smoke-free cigarettes mitigate damage due to combustion (although companies admit that they cannot confirm that these products are harm-free). Legitimacy is a dynamic concept; evidently, for these companies, there is a paradigm shift on the horizon dictated by, among other issues, growing problems of legitimacy and the sinking profitability of the markets in which they operate. Hence, the transactional–more than the transformational–need to find different value propositions, product categories, customer segments, and, ultimately, new revenue streams. From an accountability perspective, it is interesting to comprehend how these business model considerations, although available for the review of interested stakeholders and shareholders, are generally not presented in highly visible parts of the reports.

Moreover, in terms of report structure and content visibility, the data collected through our content analysis confirmed that the vast majority of the images were used to communicate a positive message and build a perception of trust around the organization that published the report. Smiling, confident customers and clean and well-lit production facilities and resorts are examples of common visual elements depicted in the reports. This finding is in line with legitimacy theory and the intention to use reporting to promote a sense of legitimacy among stakeholders (Invernizzi et al., 2022). Despite the “addiction industry” background and the glaring negative externalities of certain products and services, our content analysis–unsurprisingly, considering the literature on the subject–confirmed that there were no reports with at least one image with a negative meaning (e.g., connected to irresponsible use or health consequences).

Based on these premises, during the content analysis, we also manually collected the most salient visual stimuli (i.e., the most thought-provoking report pages, with visual and textual content) concerning the subject of addiction, the admission of responsibility, the promotion of responsible consumption, the transition to new, less legitimacy-threatening products and models of business, the implemented remedial actions, and a more dialogic relationship with stakeholders. These stimuli collected during the content analysis would inform the construction of the experiment about stakeholder perceptions that constituted Study 2, as illustrated in the next section.

## Study 2: Experiment on Effectiveness and Hypocrisy Perception

### Methodology and Data Collection

Study 2 is an online between-subject experiment. It tests the effect of different CSR strategies (preventive vs. remedial) on organizational legitimacy and the mediating role of perceived hypocrisy and perceived effectiveness. The study was conducted in December 2021. The respondents were recruited on Prolific Academic and thus received a monetary incentive. The respondents were prescreened following two criteria. Specifically, we selected people of UK nationality to be consistent with the companies of the content analysis. We also required that the respondents speak English as their native language to ensure comprehension of the questionnaire and the stimuli. The respondents were randomly assigned to one of the two conditions (preventive vs. remedial CSR activity) and were asked to read a partial SES report page from a fictitious company. We empirically tested the effect of the CSR strategy across two different contexts, namely, the alcohol and gambling contexts, to emphasize the generalizability of the CSR strategy categorizations. Thus, by testing the CSR strategies in these dissimilar contexts, we were able to enhance the external validity of our main effect. We opted not to include a real company because the resulting associations could have produced distortions that would undermine our findings (Bellucci et al., 2021a, 2021b). However, we built on our content analysis to present a realistic scenario. We manipulated the two variables via the contents of the SES report. The fictional companies were presented in the cover story as follows:

Greenwich is a UK-based international company active since 1940 in the alcohol industry. Last year Greenwich published a corporate social responsibility report that indicates some social responsibility strategies implemented by the company.

Greenwich is a UK-based international company active since 1940 in the gambling industry. Last year Greenwich published a corporate social responsibility report that indicates some social responsibility activities implemented by the company.

Then, we presented the SES report page to the respondents (see Appendix). The preventive CSR strategy scenario regarding the alcohol industry was manipulated as follows:

We are investing in research to find alternative solutions to alcohol, such as alcohol-free beer. This can prevent health damages due to alcohol consumption that affect the health of many people in the UK.

In the remedial CSR strategy, the respondents read the following:

We are investing in research on cancer to limit the damages of alcohol consumption. This can help cure health damages due to alcohol consumption that affect the health of many people in the UK.

The preventive CSR strategy scenario regarding the gambling industry was manipulated as follows:

We invest in research and lead in the development of practice standards to prevent irresponsible gameplay and addiction. This can prevent serious damages due to gambling addiction that annually involves many people in the UK.

In the remedial CSR strategy, the respondents read the following:

We invest in research and lead in the development of practice standards to cure gaming disorders. This can cure serious damages due to gambling addiction that annually involves many people in the UK.

Then, the participants were asked to complete measures of perceived hypocrisy, perceived effectiveness and organizational legitimacy using a 7-point Likert validated scale. The participants provided ratings of perceived hypocrisy (*α* = 0.855) using a 6-item measure provided by Wagner et al. (2009). Perceived effectiveness (*α* = 0.887) was measured by adapting the 3-item scale presented by Lee et al. (2018). Finally, organizational legitimacy (*α* = 0.813) was assessed using the 3-item scale by Bachmann and Ingenhoff (2016) (see Table 3). We checked the manipulations by asking the respondents to indicate to what extent the CSR strategy was aimed at preventing (vs. curing) the damage to consumers’ health. Finally, we asked them to indicate their sex and age.

**Table 3:** Measures of constructs

Construct	Source	Item
Perceived effectiveness	Lee et al. (2018)	1. I think the company can make a big impact through the social responsibility activity explained in the report
		2. There is a great need for the contribution of the company to the issue
		3. The social responsibility activity presents a significant opportunity for the company to make a difference
Perceived hypocrisy	Wagner (2009)	1. The company acts hypocritically
		2. What the company says and does are two different things
		3. The company pretends to be something that it is not
		4. The company does exactly what it says.^a^
		5. The company keeps its promises.^a^
		6. The company puts its words into action.^a^
Organizational legitimacy	Bachmann and Ingenhoff (2016)	1. It seems to me that the company acts consistently with socially accepted norms and values
		2. In my opinion, the company seems to be a legitimate company
		3. I have the impression that the company complies with social and ecological standards

^a^Item responses were reverse coded

## Results

Before we conducted the actual experiment, we assessed the manipulations through an online pilot study on Prolific Academic. The pretest regarding the stimuli related to the alcohol company involved 101 participants (Mage = 39; 60% female). The participants were randomly assigned to one of two treatment conditions (preventive vs. remedial CSR strategy). Then, they were asked to answer two questions. They rated the extent to which the CSR strategy was aimed at preventing damage to consumers’ health and curing damage to consumers’ health. The results confirmed the intended effect of our manipulations. Participants in the preventive CSR strategy expressed a significantly greater degree of belief that the CSR strategy described in the CSR report page was aimed at preventing health issues (Mpreventive = 4.24, Mremedial = 3.36, *p* = 0.007).

90 participants (Mage = 33; 51% female) took part in the pretest to check the manipulation in the context of the gambling industry. We filtered the participants by excluding those who had participated in the pretest related to the alcohol industry. Participants in the preventive CSR strategy expressed a significantly greater belief that the CSR strategy described in the CSR report page was aimed at preventing health issues (Mpreventive = 4.46, Mremedial = 3.48, *p* = 0.005).

**Fig. 2:**
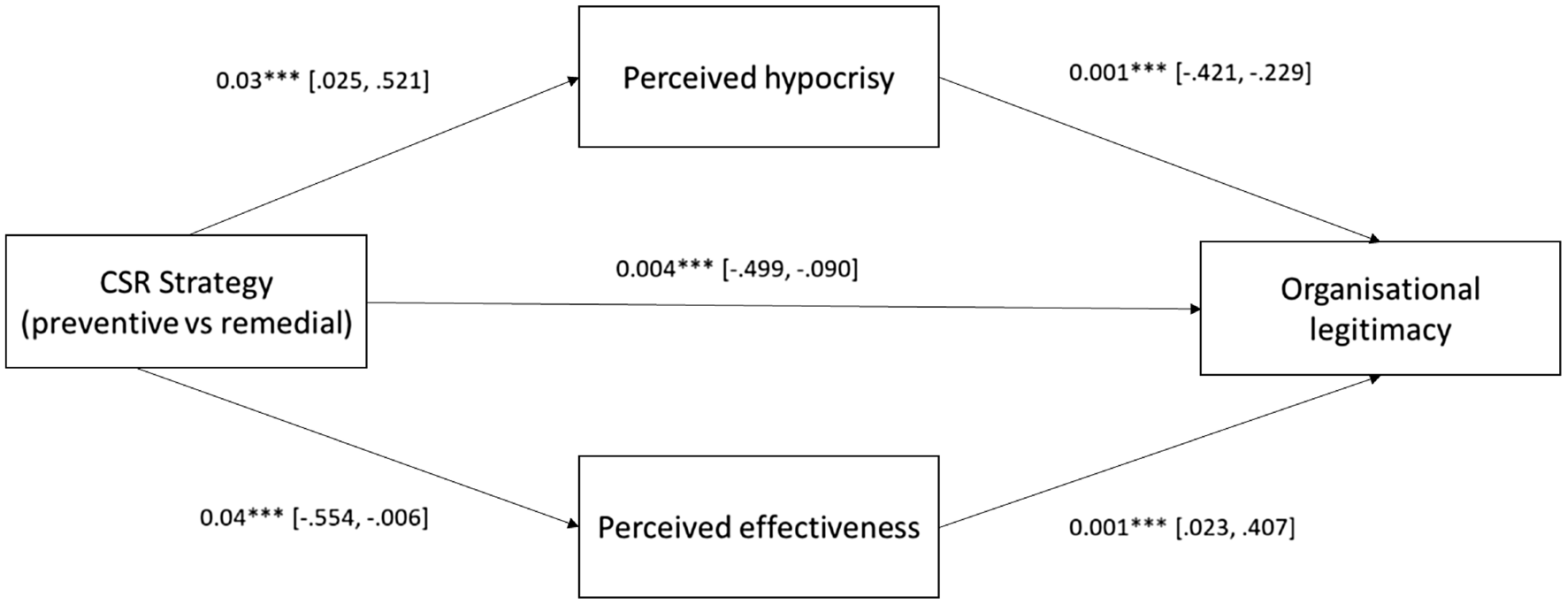
The mediating role of perceived effectiveness and perceived hypocrisy

A total of 331 participants took part in the main experiment for Study 2 (Mage = 37; 55% female). We excluded participants who had participated in the previous pretests from this study. We first checked for the manipulations. Participants in the preventive CSR strategy expressed a significantly greater belief that the CSR strategy described in the CSR report page was aimed at preventing health issues (Mpreventive = 3.97, Mremedial = 3.62, *p* = 0.048). One-way ANOVA was performed to test whether the type of CSR strategy (preventive vs. remedial) had a direct effect on perceived organizational legitimacy (H1). The results showed a significant difference between the two conditions (*F* = 14.4 *p* < 0.01), thus supporting H1. Participants who read the preventive strategy statement had a higher perception of the legitimacy of the company (Mpreventive = 4.69) than those presented with the remedial strategy (Mremedial = 4.22). We anticipated that consumers would attribute more legitimacy to companies adopting a preventive CSR strategy because it activates different levels of perceived hypocrisy and perceived effectiveness of the strategy (H2 and H3). Therefore, we tested a parallel mediation model using the PROCESS macro (Model 4; *n* = 10,000) by Hayes (2017), with the type of CSR strategy as the independent variable and perceived hypocrisy (mediator 1) and perceived effectiveness (mediator 2) as mediators. The type of CSR strategy had a significant effect on perceived hypocrisy (*β* = 0.27, *SE* = 0.12, *t* = 3.23, *p* < 0.03) and perceived effectiveness (*β* = − 0.28, *SE* = 0.13, *t* = − 2.01, *p* = 0.04). In turn, perceived hypocrisy (*β* =  −  0.32, *SE* = 0.04, *t* =  − 6.69, *p* < 0.001) and perceived effectiveness (*β* = 0.32, *SE* = 0.04, *t* = 7.29, *p* < 0.001) had a significant effect on perceived legitimacy, confirming H2 and H3. The results are shown in Fig. 2. We ran an additional robustness check test (Viglia et al., 2018) to verify the robustness of our findings across the three industries and the six countries. Specifically, we replicated Study 2 on Prolific Academic involving 169 (Mage = 39; 65% female) additional respondents from the 6 countries and presenting participants with a tobacco company’s CSR report. The preventive CSR strategy scenario regarding the tobacco industry was manipulated as follows:

“We want to deliver a smoke-free future. We will continue to responsibly increase awareness of smoke-free products”.

In the remedial CSR strategy, the respondents read the following:

*“We implement measures aimed at addressing the adverse impacts of our business. We will continue to support research that cures health damage caused by cigarettes”.*

The manipulation check confirmed the reliability of our manipulations (Mpreventive = 4.19, Mremedial = 2.34, *p* < 0.001). The reliability analysis of the scales showed a Cronbach’s Alpha higher than 0.75 for organizational legitimacy (*α* = 0.843), perceived hypocrisy (*α* = 0.893) and perceived effectiveness (*α* = 0.790). A one-way ANOVA showed a significantly different effect of the type of CSR strategy (preventive vs. remedial) on perceived organizational legitimacy (*F* = 18.4 *p* < 0.01), thereby supporting H1. The participants in the preventive strategy scenario had a higher perception of legitimacy (Mpreventive = 4.74) than those presented with the remedial strategy (Mremedial = 3.97). The parallel mediation analysis (Model 4; *n* = 10,000) was also significant. The type of CSR strategy had a significant effect on both perceived hypocrisy (*β* = 0.34, *SE* = 0.17, *t* = 1.98, *p* < 0.05) and perceived effectiveness (*β* = − 0.36, *SE* = 0.18, *t* = − 1.94 *p* = 0.05). In turn, perceived hypocrisy (*β* = − 0.46, *SE* = 0.06, *t* =  − 7.44, *p* < 0.001) and perceived effectiveness (*β* = 0.36, *SE* = 0.05, *t* = 6.36, *p* < 0.001) had a significant effect on perceived legitimacy, confirming H2 and H3.

## Discussion and Conclusions

CSR reports are a convenient instrument for companies to influence the perceptions of their stakeholders (Invernizzi et al., 2022) and improve their legitimacy (Ahn & Park, 2018). This opportunity to use nonfinancial disclosure to create social approval is ambiguous for companies marketing products or services that potentially generate addiction in consumers.

Although “addiction industry” companies have a legal license to operate, they do not necessarily receive approval from society to justify their potentially harmful products and services (Dhandhania & O’Higgins, 2021). These companies are generally delegitimized by stakeholders, as they manufacture and distribute products or services that create addiction in consumers, thus making money from exploiting human vices and health. Despite the controversial nature of “addiction industry” companies and the contentious use of CSR reports, recent studies have proposed the possibility of these organizations enhancing their reputation and mitigating their negative image through CSR reports under certain circumstances (Oh et al., 2017).

In this paper, we focused on the CSR strategies implemented and disclosed by companies that sell addictive products and services, and we observed their effect on stakeholders’ perceptions. In particular, we assessed how companies disclosed their CSR strategies in nonfinancial reports (Study 1) and how the type of CSR strategy affected the organizational legitimacy perception of stakeholders (Study 2). While Study 1 confirmed that “addiction companies” implement and disclose both preventive and remedial strategies, in Study 2, we compared such strategies and showed that they affect stakeholders’ perceptions differently.

Specifically, in Study 1, we analyzed a large set of companies operating in the addiction sectors (tobacco, alcohol, and gambling), and we investigated the actions they took to counteract the harmful effects of their products and services on consumers. Initially, we found that only 49 out of 136 companies published a sustainability or integrated report in 2021: we think this is an interesting result in itself. The fact that many of the “addiction companies” opted not to publish a sustainability report could be explained by the desire not to make specific disclosures that are not mandatory, especially considering the growing negative opinions generated by the increased awareness of the physical and psychological health impacts of certain ranges of products and services such as traditional cigarettes or gambling. Proportionally, European companies appear more likely to prepare a report with social or environmental content: this could be an effect of the European Non-Financial Reporting Directive (Barbu et al., 2022; Krasodomska et al., 2021; Mio et al., 2020). A further interpretation at the sector level is that, proportionally, many of the companies operating in the alcohol sector are listed on European markets and that alcohol companies have been shown to be more transparent and more inclined toward social and environmental disclosure than tobacco companies and especially gambling companies.

Moreover, we found that only a few companies explicitly referred to the addictive nature of their products and services while indirectly admitting it by encouraging consumers to moderate their consumption. However, our content analysis highlighted many thought-provoking, sector-specific differences in how companies try to maintain their legitimacy through preventive or remedial strategies. While tobacco companies are more prone to disclose information about the negative consequences of their products, alcohol and gambling companies prefer to adopt a different type of disclosure oriented toward encouraging responsible consumption, which deflects responsibility from the producer to the consumers. Companies in the alcohol and gambling sectors are reluctant to disclose their preventive responsibility for the negative effects of their products but instead are proactive in showing commitment to the implementation of remedial actions. In contrast, tobacco companies avoid the disclosure of remedial actions (which could appear deceitful in light of the dissonance between talk and action), show greater willingness to clearly take responsibility in the reports for the negative effects of the consumption of their products, and often focus their disclosure on the development of different business models built on “risk-reduced” products. The scarce preventive communications of the alcohol and gambling industries may be due to how the negative consequences of their respective products are less prominent and acknowledged; thus, the communication of preventive strategies could emphasize the potential harm of alcohol and gambling to consumers. However, this choice could negatively affect stakeholders’ perceptions in terms of hypocrisy, effectiveness, and, in turn, legitimacy because the related remedial actions are implemented after the damage is done.

Drawing from this overview, in Study 2, we compared preventive vs. remedial CSR strategies and tested whether the former strategies lead to more positive assessment in terms of hypocrisy perception, action effectiveness, and perceived legitimacy. An online experiment assessed stakeholders’ perceptions of organizational legitimacy depending on the CSR strategy adopted by the company (preventive vs. remedial). The findings showed that the preventive strategy was preferable to the remedial strategy, as it reduced hypocrisy perceptions and increased the perceived effectiveness of the strategy, enhancing organizational legitimacy. This evidence is consistent with studies in public policy that have supported the development of prevention and early intervention over remedial approaches (Kennedy, 2020).

We believe that this research makes four main contributions to the literature.

First, this study contributes to the strand of literature investigating the use of CSR reporting to build organizational façades and build/maintain/restore organizational legitimacy. We provide original and up-to-date empirical evidence of how companies in the tobacco, alcohol and gambling sectors apply a mix of different but complementary strategies to maintain their legitimacy through their CSR reports. This legitimacy and their profit opportunities are gradually being jeopardized by a public and medical discussion increasingly oriented toward condemning the negative consequences that can arise (or certainly do arise) from the use of these companies’ products. In particular, our study has the merit of being the first to focus on reporting on the subject of addictions and related responsibilities. From an accountability perspective, this is particularly important because previous studies have focused on the issue of “sin” (Cai et al., 2011) or “harm” (Lindorff et al., 2012) industries, while the problem of addiction is even more difficult for companies to report and legitimate due to the long-term negative consequences for users. The “addictive” aspect of some industries also presents new problematizations for the business ethics field. By defining the characteristics of industries, we provide business ethics scholars with a new perspective for analyzing the legitimacy of businesses. For instance, while tobacco, alcohol and gambling are condemned by the majority of consumers, other industries that create harmful addictions are considered more legitimate. The highly processed food sector, for example, induces repetitive behavior that some people find difficult to quit—eating processed food–which can lead to addiction (Schulte et al., 2018). Some authors from the gray literature even suggest that processed foods–such as cheeseburgers or ice cream—are not only addictive but that “they can be even more addictive than alcohol, tobacco and drugs” (O’Connor, 2021), causing dangerous disorders. Hence, considering the addictive dimension of businesses can help in exploring the legitimacy of businesses in greater depth and advancing the investigation of the business ethics field. Against these considerations, our discussion of several reporting patterns in the different “addiction industries” provides a useful multidisciplinary contribution for practitioners and scholars interested in addressing the issues associated with the organizational legitimacy of companies operating in controversial sectors. Indeed, the addictive aspect of stigmatized industries’ activity can open novel considerations for accounting (Sharma & Song, 2018), organization (Reuber & Morgan-Thomas, 2019), marketing (Abdollahi et al., 2022), and management (Ashforth, 2019) research. For example, investigating the addiction caused by processed foods can contribute to the scientific dialogue on ambivalence (“simultaneously positive and negative orientations toward an object”; Ashforth, 2019, p. 27) and legitimacy in management studies, as it uncovers a negative–and somewhat veiled–aspect of a legitimate industry.

Second, this research identifies two processes that lead stakeholders in their evaluation of organizational legitimacy. Previous studies have demonstrated the importance of CSR disclosure in creating organizational legitimacy (Bellucci et al., 2021a; Chauvey et al., 2015); however, little is known about the cognitive mechanisms leading to legitimacy assessment. We find that stakeholders delegitimize companies when they perceive them to be hypocritical. Indeed, misalignment between companies’ claims and actions shows the pursuit of economic goals in a socially unacceptable manner. In addition, the perceived inefficacy of CSR actions has negative effects on legitimacy assessment. Indeed, when the CSR strategy does not produce the intended result for the beneficiaries, stakeholders do not trust the claimed efforts of the company to compensate for the negative impact of its business. Consequently, the disclosed CSR action appears to be merely a means of cleaning the corporate reputation. This mechanism leads to reduced legitimacy. This evidence advances the knowledge of perceived hypocrisy and effectiveness in CSR disclosure, providing a novel theoretical explanation of legitimacy perception. Accordingly, companies should be careful in the selection of their CSR strategies and communicate their effectiveness clearly and credibly.

Third, this research enriches the knowledge of organizational legitimacy by observing the perceptual effects of preventive vs. remedial strategies. Although the preferability of preventive actions has been recognized in popular culture and applied to social care and public policy interventions, we investigate its role in determining companies’ legitimacy. We bring this assertion to the context of CSR disclosure, and we study its effect on stakeholders’ perceptions. The use of nonfinancial reports by addiction industry companies is a crucial yet controversial instrument for communicating with stakeholders and sharing responsible practices with them (Dhandhania & O'Higgins, 2021). It is not clear which elements of CSR disclosure help these companies appear legitimate. In this paper, we propose that CSR strategies are a potential determinant of stakeholders’ perceptions and suggest that companies prevent the negative effects of their products and services instead of curing the harm that they cause to consumers.

Fourth, our findings contribute to the literature on legitimacy and organized hypocrisy with an empirically grounded discussion of how a company’s behavior in terms of reporting can help build an organizational façade rooted in demarketing strategies (Comm, 1998) and business model development. On the one hand, our study substantiates that, for businesses, the most effective legitimization strategy concerning harm and addiction is to take responsibility and initiate preventive strategies; this is also reflected in managerial and reporting choices that invite consumers to reduce consumption of a certain product. However, these demarketing activities are often counterbalanced by the creation of new lines of products–for example, non-alcoholic beers or electronic cigarettes–towards which consumer preferences are redirected. What is at stake, therefore, is that this attention to organizational legitimacy and the management of an organizational façade go hand-in-hand with more or less veiled corporate hypocrisy and the enduring willingness to restore eroding profits. Further research could build on this study to expand, from both an accounting (contents and quality of corporate CSR reporting) and marketing (effect of communication on stakeholders’ perceptions) perspective, the understanding of the tools that companies use to manage their organizational legitimacy.

As our research is limited to three addiction industries, further studies could focus on other controversial topics and sectors, such as companies in other potentially addictive “vice” industries (e.g., adult entertainment, online gaming and social media providers), product-related screening categories used to filter companies in ESG or SRI investing (e.g., nonrenewable energy and weapons), or conduct-related controversies in diversity practices, disability and inclusiveness that violate the UN Global Compact Principles. Our research is also limited to a single year of operation while further empirical studies could provide diachronic evidence to foster an understanding of the evolution of disclosure in addiction industries. In particular, an interesting development would be to examine how firm disclosures in the addiction industries differ before, during, and after the Covid-19 pandemic.

Moreover, our study has the limitation of focusing only on Western markets; further studies investigating the role of CSR reporting for companies in controversial sectors operating in Eastern countries would contribute to the understanding of how organizational legitimacy, CSR reporting and stakeholders’ perceptions can be influenced by different social, political, institutional, cultural and religious contexts. Indeed, studies on business ethics have reported different results in Western and Eastern societies (Chen & Moosmayer, 2020). This inconsistency challenges the generalizability of our findings in different cultural contexts. Finally, this paper aimed to investigate how stakeholders perceive addictive companies, and the effectiveness of their actions, comparing preventive vs. remedial actions. However, we believe that it would be interesting to conduct more in-depth investigations of each of the preventive and remedial social responsibility strategies to understand how they influence the organizational legitimacy of addictive businesses. For instance, we found that some companies promote responsible modes of consumption in their reports such as “when you drive, never drink”. In this case, the company “delegates” part of the responsibility to consumers, legitimizing the use of addictive products/services for those who are careful and responsible. Thus, we ask, how do consumers perceive these types of claims? Does this sharing of responsibility make the company appear opportunist? Or, on the contrary, does the company appear more legitimate when it transfers the responsibility to consumers? We believe that these could be fascinating avenues for further research on legitimacy and controversial industries.

## Data Availability

The data that support the findings of this study are available from the authors upon reasonable request.

## Appendix

### Appendix 1

See Table 4

**Table 4:** Quantitative results from the content analysis of the disclosure of companies in addiction industries

	General		Strategy: preventive						Strategy: remedial				Additional elements			
	Addiction issues		Responsible consumption		Demarketing		Changes in business model		Assumption of responsibility		Remedial actions		Dialogic stakeholder engagement		Negative images	
	*All addiction industries*															
Reports that mention this aspect (in plain sight)	9	18.37%	12	24.49%	11	22.45%	20	40.82%	8	16.33%	12	24.49%	15	30.61%	0	0.00%
Reports that mention this aspect (not in plain sight)	4	8.16%	26	53.06%	5	10.20%	2	4.08%	4	8.16%	16	32.65%	23	46.94%	0	0.00%
**Reports that mention this aspect**	**13**	**26.53%**	**38**	**77.55%**	**16**	**32.65%**	**22**	**44.90%**	**12**	**24.49%**	**28**	**57.14%**	**38**	**77.55%**	**0**	**0.00%**
Reports that omit this aspect	36	73.47%	11	22.45%	33	67.35%	27	55.10%	37	75.51%	21	42.86%	11	22.45%	49	100%
*Total reports analyzed*	*49*	*100%*	*49*	*100%*	*49*	*100%*	*49*	*100%*	*49*	*100%*	*49*	*100%*	*49*	*100%*	*49*	*100%*
	*Alcohol industry*															
Reports that mention this aspect (in plain sight)	6	25.00%	7	29.17%	5	20.83%	13	54.17%	4	16.67%	8	33.33%	6	25.00%	0	0.00%
Reports that mention this aspect (not in plain sight)	0	0.00%	11	45.83%	2	8.33%	1	4.17%	1	4.17%	8	33.33%	11	45.83%	0	0.00%
**Reports that mention this aspect**	**6**	**25.00%**	**18**	**75.00%**	**7**	**29.17%**	**14**	**58.33%**	**5**	**20.83%**	**16**	**66.67%**	**17**	**70.83%**	**0**	**0.00%**
Reports that omit this aspect	18	75.00%	6	25.00%	17	70.83%	10	41.67%	19	79.17%	8	33.33%	7	29.17%	24	100%
*Total reports analyzed*	*24*	*100%*	*24*	*100%*	*24*	*100%*	*24*	*100%*	*24*	*100%*	*24*	*100%*	*24*	*100%*	*24*	*100%*
	*Gambling industry*															
Reports that mention this aspect (in plain sight)	2	11.76%	4	23.53%	2	11.76%	2	11.76%	3	17.65%	4	23.53%	6	35.29%	0	0.00%
Reports that mention this aspect (not in plain sight)	1	5.88%	12	70.59%	0	0.00%	0	0.00%	0	0.00%	7	41.18%	8	47.06%	0	0.00%
**Reports that mention this aspect**	**3**	**17.65%**	**16**	**94.12%**	**2**	**11.76%**	**2**	**11.76%**	**3**	**17.65%**	**11**	**64.71%**	**14**	**82.35%**	**0**	**0.00%**
Reports that omit this aspect	14	82.35%	1	5.88%	15	88.24%	15	88.24%	14	82.35%	6	35.29%	3	17.65%	17	100.00%
*Total reports analyzed*	*17*	*100%*	*17*	*100%*	*17*	*100%*	*17*	*100%*	*17*	*100%*	*17*	*100%*	*17*	*100%*	*17*	*100%*
	*Tobacco industry*															
Reports that mention this aspect (in plain sight)	1	12.50%	1	12.50%	4	50.00%	5	62.50%	1	12.50%	0	0.00%	3	37.50%	0	0.00%
Reports that mention this aspect (not in plain sight)	3	37.50%	3	37.50%	3	37.50%	1	12.50%	3	37.50%	1	12.50%	4	50.00%	0	0.00%
**Reports that mention this aspect**	**4**	**50.00%**	**4**	**50.00%**	**7**	**87.50%**	**6**	**75.00%**	**4**	**50.00%**	**1**	**12.50%**	**7**	**87.50%**	**0**	**0.00%**
Reports that omit this aspect	4	50.00%	4	50.00%	1	12.50%	2	25.00%	4	50.00%	7	87.50%	1	12.50%	8	100%
*Total reports analyzed*	*8*	*100%*	*8*	*100%*	*8*	*100%*	*8*	*100%*	*8*	*100%*	*8*	*100%*	*8*	*100%*	*8*	*100%*

### Appendix 2

Extract of one page of the report read by the participants.
